# Ophthalmic Artery Aneurysm in a Cadaver: Case Report

**DOI:** 10.7759/cureus.2818

**Published:** 2018-06-16

**Authors:** Dominic Dalip, Joe Iwanaga, Marios Loukas, Rod J Oskouian, R. Shane Tubbs

**Affiliations:** 1 Seattle Science Foundation, Seattle, USA; 2 Anatomical Sciences, St. George's University, St. George's, GRD; 3 Neurosurgery, Swedish Neuroscience Institute, Seattle, USA; 4 Neurosurgery, Seattle Science Foundation, Seattle, USA

**Keywords:** ophthalmic artery, aneurysm, ophthalmoplegia, embolization

## Abstract

The ophthalmic artery arises from the supraclinoid segment of the internal carotid artery (ICA) and enters the orbit through the optic canal. It perfuses the orbit and the orbit apparatus. Ophthalmic artery aneurysms (OAA) account for 0.5% to 11% of all intracerebral aneurysms. Patients are usually asymptomatic but, in some cases, patients can present with ophthalmoplegia and total blindness if these aneurysms rupture. Aneurysms are usually diagnosed using computed tomography (CT) angiography but can also be seen on magnetic resonance imaging (MRI) and four-vessel digital subtraction angiography. Treatment of OAA entails either surgical or endovascular approaches with the mortality rate for surgical treatment as high as 25%, whereas embolization with balloon therapy is deemed safer with mortality rates around 9%. Recent techniques of embolization coiling have had even better results.

## Introduction

**Figure 1:**
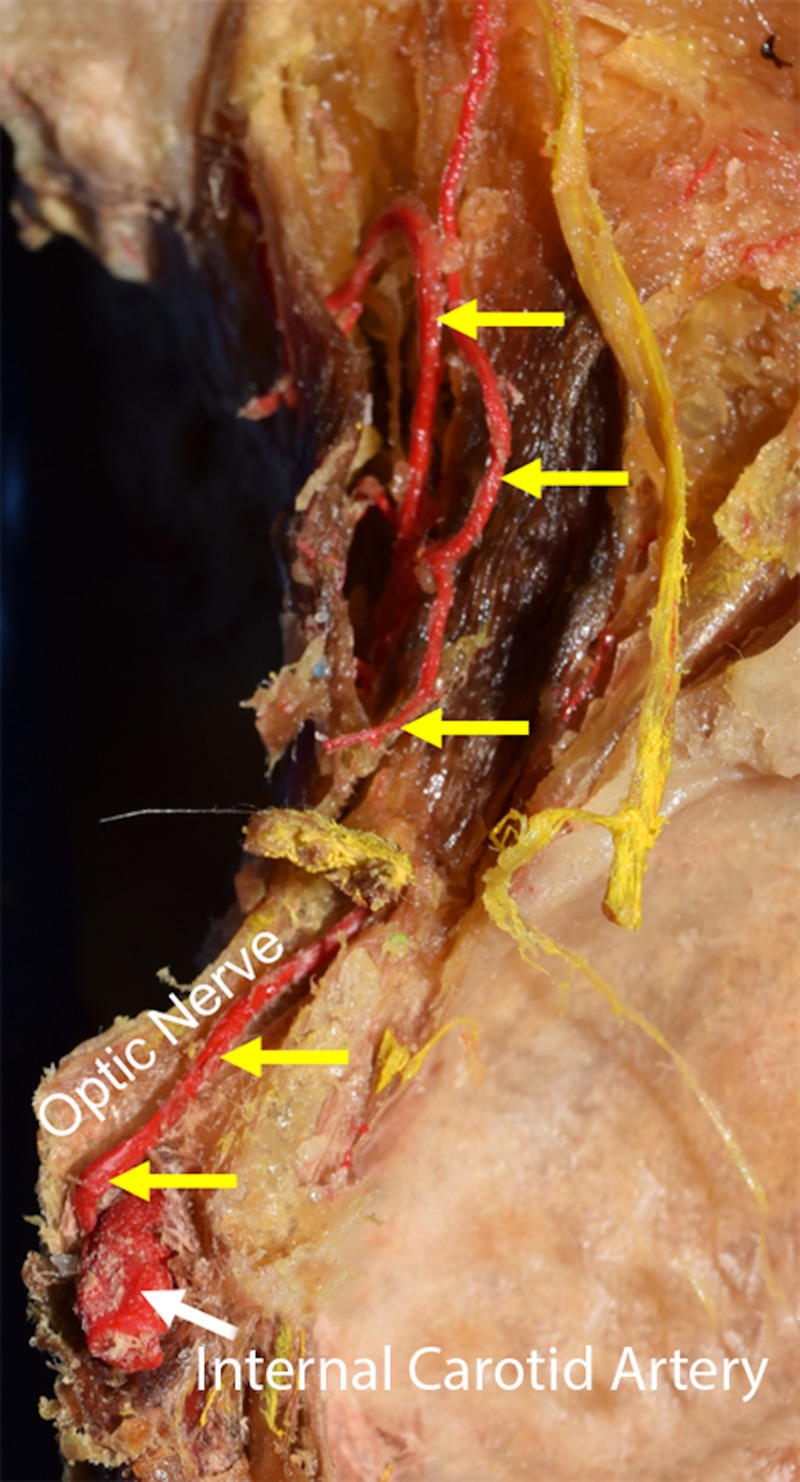
Course of the right optic artery arising from the internal carotid artery in normal anatomy (yellow arrows) The optic nerve is reflected medially.

Arising from the internal carotid artery (ICA), the ophthalmic artery is vital to perfusing the orbit and optic apparatus (Figure 1). Therefore, any compromise can have profound clinical manifestations. Ophthalmic artery aneurysms (OAA) account for 0.5% to 11% of all intracerebral aneurysms [1]. Sometimes these aneurysms can be large enough to extend proximally and involve the ICA [1]. True OAA are extremely rare; hence, there are not many published reports on this topic. Patients are usually asymptomatic but, in some cases, patients can present with ophthalmoplegia and total blindness if these aneurysms rupture. OAA can become large enough to compress nearby structures in the orbit, which can lead to diplopia, optic nerve atrophy, and exophthalmos [2]. OAA should be recognized as a potential cause of these symptoms and care should be taken in deciding the management for these patients. Treatment involves common carotid artery ligation, ICA occlusion, extracranial-intracranial artery bypass, balloon occlusion, coiling or stenting [1]. Surgical management of the OAA carries the risk of hemorrhage, stroke, and visual disturbances [1,3].

Here, we report an interesting case of an incidentally found ophthalmic artery aneurysm in a cadaver. This unique case offers the opportunity to view such pathology in regard to its anatomical relationships.

## Case presentation

**Figure 2:**
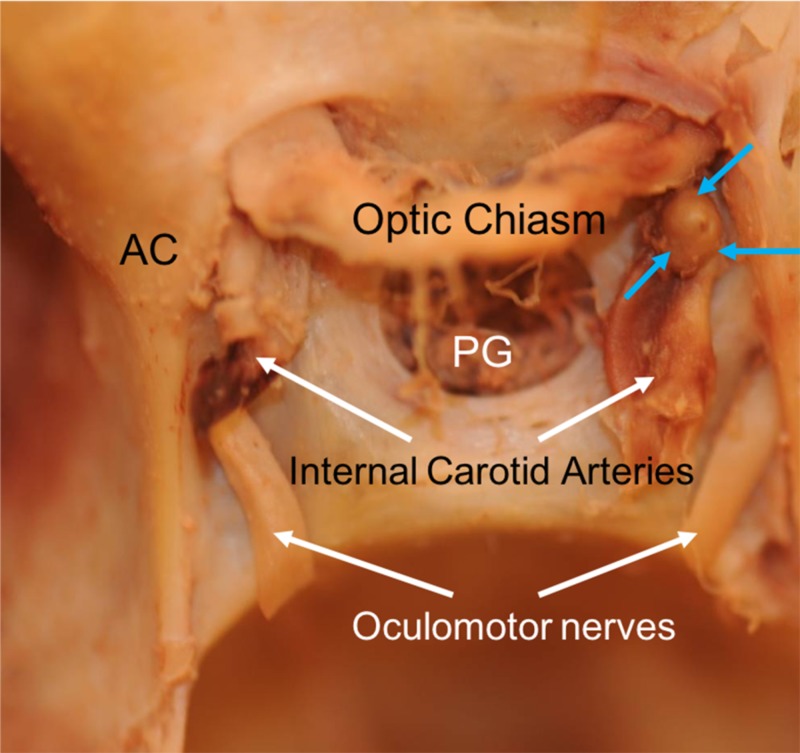
Right ophthalmic artery aneurysm (blue arrows) as seen in the current report

The ophthalmic artery arises from the ICA and is a major artery that perfuse the orbit and its structures (Figure 1). During routine dissection of the skull base in an adult formalin-fixed male cadaver who died at the age of 69, we encountered a right OAA (Figure 2). The specimen was a Caucasian with cardiac arrest as the cause of death. No other known medical issues existed. No other intracranial pathology or anatomical variations were noted. The related structures around the right OAA were the optic nerve superiorly, the anterior clinoid (AC) process anterolaterally, and the anterior petroclinoid fold laterally. The OAA measured 3 x 2.5 mm and slightly elevated the overlying optic nerve and seemed to efface the medial wall of the supraclinoid part of the internal carotid artery from which it arose. Thrombus filled the aneurysm and both the OAA and adjacent ICA were atherosclerotic.

## Discussion

The ophthalmic artery arises from the supraclinoid segment of the ICA and enters the orbit through the optic canal [4]. The ophthalmic artery then branches into the central retinal, lacrimal, ciliary, anterior and posterior ethmoidal artery, which gives most of the blood supply to the orbit. OAA are challenging for both neurosurgeons and interventional radiologists because of the fragility and small nature of the vessel, relationship to vital structures, location to the anterior clinoid process, and the risk of visual disturbances in patients. Aneurysms are usually diagnosed using computed tomography (CT) angiography, but can also be seen on magnetic resonance imaging (MRI) and four-vessel digital subtraction angiography [5]. In the literature, there is a case where a 27-year-old female complained of pulsating bitemporal headaches and denied any other neurological problems such as vision changes, altered mental status, or any sensory or motor deficits [6]. The patient had these headaches for a couple of months with an intensity of 10/10 on the pain scale. The headache pattern and distribution did not match any of the typical types of headaches. CT angiography was performed and revealed a 3-4 mm left sided unruptured OAA. After treatment with endovascular coiling, the patient’s symptoms resolved.

Treatment of OAA entails either surgical or endovascular approaches with the mortality rate for surgical treatment as high as 25%, whereas embolization with balloon therapy is deemed safer with mortality rates around 9%. Recent techniques of embolization coiling have had even better results [5]. However, if the aneurysm is less than 7 mm in diameter and the patient is asymptomatic without any risk factors, then the patient should be managed conservatively [6]. For large unruptured OAA, it is better to surgically clip the artery than to coil because it allows for aneurysmal sac decompression which can obliterate the compression of the optic nerve [1]. However, if a symptomatic patient has a small OAA, then the endovascular coiling approach should be used. The literature also mentions a case where both surgical clipping and endovascular temporary carotid balloon occlusion were performed in treating a 29-year-old female with a large, broad-based, right OAA [7].

## Conclusions

The OAA was found during our routine dissection of a cadaver. To our knowledge, this is the first report of an OAA in a cadaveric specimen and such a case offers a unique window into this pathology’s significant anatomical relationships.
